# Supplementary material for: A conserved role of the duplicated Masculinizer gene in sex determination of the Mediterranean flour moth, Ephestia kuehniella
Source: PLoS Genet. 2021 Aug 2;17(8):e1009420. doi: 10.1371/journal.pgen.1009420 (PMC8360546; doi:10.1371/journal.pgen.1009420)
Supplement: S1 Table — An unpaired two-tailed t-test for unequal variances was used. Expression levels significantly differing from each other between sexes (P < 0.05) are indicated in bold. (PDF) [file pgen.1009420.s001.pdf]

S1 Table

Time point (hpo)	*P*-value	
	EkMasc	*EkMascB*
12	0.09913	0.1169
14	**0.01073**	**4.34e-03**
16	**5.68e-05**	**1.76e-06**
18	**0.03325**	**1.2e-04**
20	**1.05e-03**	**6.37e-03**
22	**0.02322**	**1.1e-04**
24	0.3119	0.1019
