# Supplementary material for: A conserved role of the duplicated Masculinizer gene in sex determination of the Mediterranean flour moth, Ephestia kuehniella
Source: PLoS Genet. 2021 Aug 2;17(8):e1009420. doi: 10.1371/journal.pgen.1009420 (PMC8360546; doi:10.1371/journal.pgen.1009420)
Supplement: S2 Table — Expression levels significantly differing from each other between treatments (P < 0.05) are indicated in bold. (PDF) [file pgen.1009420.s002.pdf]

S2 Table

Treatment comparison	Sex	*P*-value	
		EkMasc	*EkMascB*
siGFP – siMasc_II	Female	**0.0335**	**0.0416**
siGFP – siMasc_VII	Female	**0.0034**	**0.0090**
siGFP – siMasc_II	Male	**0.0311**	**0.0137**
siGFP – siMasc_VII	Male	**0.0300**	**0.0164**
