# Supplementary material for: A conserved role of the duplicated Masculinizer gene in sex determination of the Mediterranean flour moth, Ephestia kuehniella
Source: PLoS Genet. 2021 Aug 2;17(8):e1009420. doi: 10.1371/journal.pgen.1009420 (PMC8360546; doi:10.1371/journal.pgen.1009420)
Supplement: S3 Table — (PDF) [file pgen.1009420.s003.pdf]

S3 Table

Primer name	Primer sequence (5'- 3')	Gene	Purpose	Reference
Masc_F1	TGTCGCGAYTATGTTTGGGGW	*EkMasc/EkMascB*	Identification *EkMasc/EkMascB*<br>3' RACE-PCR (first round PCR)<br>Genetic sexing PCR	This study
Masc_R1	GGTAGCCTCCTTATTGAGCCAT	*EkMasc/EkMascB*	Identification *EkMasc/EkMascB*<br>5' RACE-PCR (second round PCR)<br>Genetic sexing PCR	This study
Adapter Primer (AP)	GGCCACGCGTCGACTAGTACTTTTTTTTTTTTTTTTT	-	RACE-PCR cDNA synthesis<br><br>First round RACE-PCR for 5'RACE	Invitrogen 3' RACE System for Rapid Amplification of cDNA Ends kit (Thermo Fisher Scientific, Waltham, MA, USA)
Abridged Universal Adapter Primer (AUAP)	GGCCACGCGTCGACTAGTAC	-	First round RACE-PCR (3'RACE)<br>Second round RACE-PCR (3'RACE and 5'RACE)	Invitrogen 3' RACE System for Rapid Amplification of cDNA Ends kit (Thermo Fisher Scientific, Waltham, MA, USA)
Masc_VII_F1	ACGYGCTGAATGATCCCGAT	*EkMasc/EkMascB*	3' RACE-PCR (second round PCR)	This study
Masc_V_R1	CGGAGGTTTGGTAGTGTCAAT	*EkMasc/EkMascB*	5' RACE-PCR (cDNA primer)	This study
Masc_IV_R1	ACAGGCCTTGATGGTGAAT	*EkMasc/EkMascB*	5' RACE-PCR (first round PCR)	This study
Masc_Sb_F	TGTTTCAGGTCCAAGCAATGT	*EkMasc/EkMascB*	Southern blot	This study
Masc_Sb_R	TCAGTCTTACTCGCAGTCTCC	*EkMasc/EkMascB*	Southern blot	This study
Masc_F_Via	ACTGAAACAACCACCTGCGA	*EkMasc/EkMascB*	qPCR Z-linkage	This study
Masc_R_VI	ATTTGTTCGCGCACCAAATGT	*EkMasc/EkMascB*	qPCR Z-linkage	This study
Ek_Ace2_F	CCCGCAACACCTAAGAGTCC	*Ace2*	qPCR Z-linkage	This study
Ek_Ace2_R	TGTACAAGTCGAGTGTGGCC	*Ace2*	qPCR Z-linkage	This study

rp49_deg_F1	ATTGACAACAGAGTSCGCAG	*rp49*	Isolation rp49	This study
rp49_deg_R1	CTGATGCTGAGCTGCTGGGC	*rp49*	Isolation rp49	This study
qrp49_F2	GGTTACGGATCAAACAAGAAGAC	*Ekrp49*	qRT-PCR	This study
qrp49_R2	GATTTCCAGCTCACGTACATTG	*Ekrp49*	qRT-PCR	This study
qMasc_F2	GCTTGGTCGGTTGAGTTTG	*EkMasc*	qRT-PCR	This study
qMasc_R2	GCCCTGTGCATTATAACCTTG	*EkMasc*	qRT-PCR	This study
qMascB_F1	GCTGGAACTTTGATGCAGAAC	*EkMascB*	qRT-PCR	This study
qMascB_R1	CCCAACCTCTTAGCGTACTTAAC	*EkMascB*	qRT-PCR	This study
Masc_bmd_qF1	TAGACATTGACACTACCAAACC	*EkMasc/EkMascB*	Splicing	This study
Masc_R_X	ACGTTGGTGGAATGACGGAA	*EkMasc/EkMascB*	Splicing	This study
qPiMasc_F1	AGAAAGCAGTGTAAACTACTTCACGAA	*PiMasc*	Splicing	This study
qPiMasc_R2	GGTCTCATTTGAACTCGAACTTCCA	*PiMasc*	Splicing	This study
CpMasc_F4	TCCGTTTCTCAACTTCGCCC	*CpMasc*	Splicing	This study
CpMasc_R2	TTAAAAGACACCGAATTATCCAGTTGT	*CpMasc*	Splicing	This study
dsx_dR_F2	CTCTAGTCCTCGTCATCCTCAA	*Ekdsx*	Determine RNAi effects on sexual development	This study
dsx_dR_R2	CGACATGCTGTACTCCTTTCTC	*Ekdsx*	Determine RNAi effects on sexual development	This study
siRNA name	siRNA sequence (5'-3')	Gene	Purpose	Reference
siMasc_II_s	CCAGGAAGAUAGAAACUGAAA	*EkMasc/EkMascB*	RNAi	This study
siMasc_II_as	UCAGUUUCUAUCUUCCUGGUU	*EkMasc/EkMascB*	RNAi	This study
siMasc_VII_s	UAGUAUGUCAAGAGAAGAUUC	*EkMasc/EkMascB*	RNAi	This study
siMasc_VII_as	AUCUUCUCUUGACAUACUAUU	*EkMasc/EkMascB*	RNAi	This study
siGFP gui-1	AUAGACGUUGUGGCUGUUGUA		RNAi (negative control)	Kiuchi et al. (2014)[a]
siGFP pas-1	CAACAGCCACAACGUCUAUUU		RNAi (negative control)	Kiuchi et al. (2014)[a]

[a] Kiuchi T, Koga H, Kawamoto M, Shoji K, Sakai H, Arai Y, et al. A single female-specific piRNA is the primary determiner of sex in the silkworm. Nature. 2014; 509(7502):633–636. doi: 10.1038/nature13315
