# Supplementary figures and images for: A conserved role of the duplicated Masculinizer gene in sex determination of the Mediterranean flour moth, Ephestia kuehniella
Source: PLoS Genet. 2021 Aug 2;17(8):e1009420. doi: 10.1371/journal.pgen.1009420 (PMC8360546; doi:10.1371/journal.pgen.1009420)

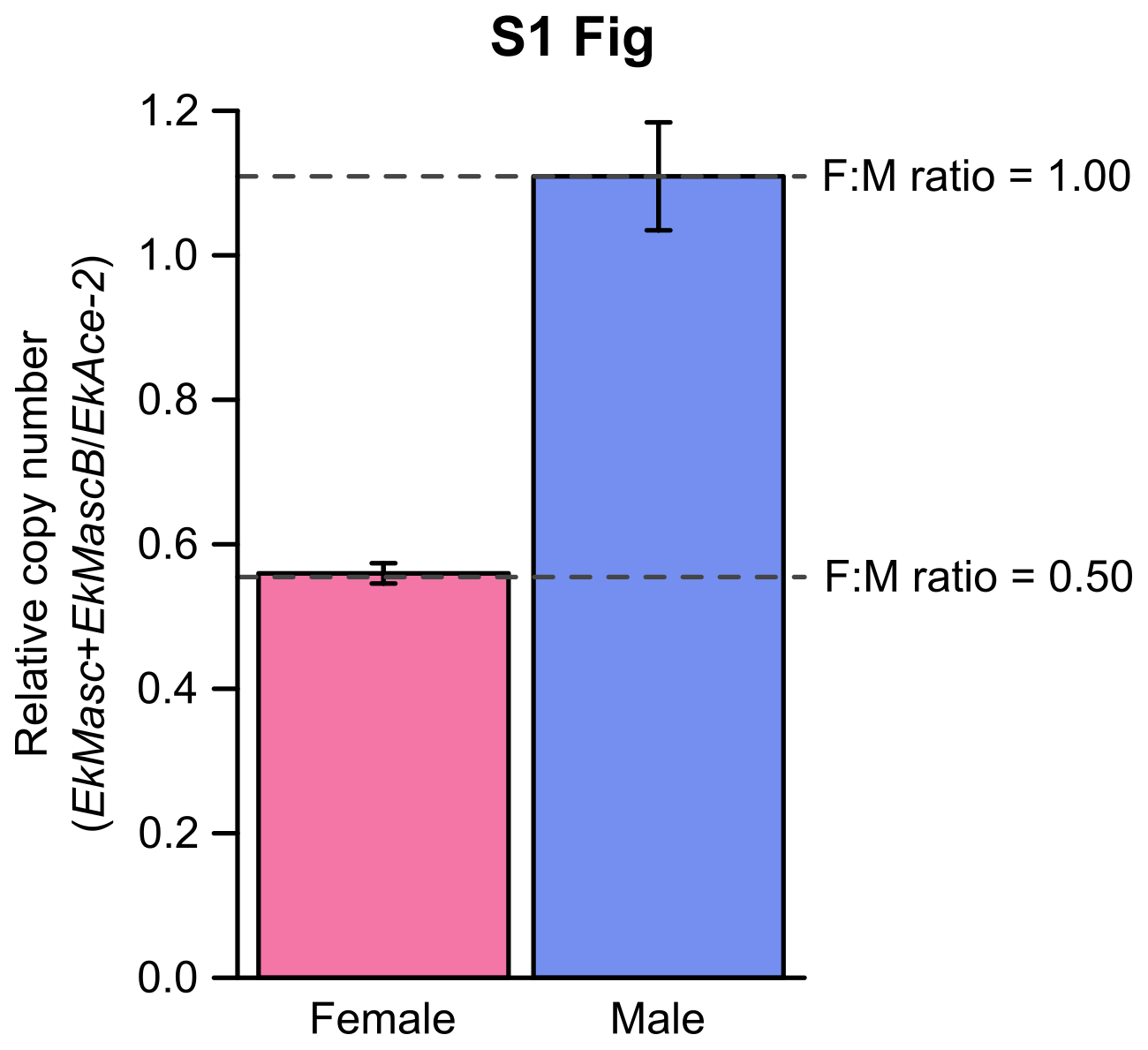

Supplement: S1 Fig — Relative copy numbers were assessed for female and male samples (n = 3 for both sexes). Indicated are hypothetical EkMasc and EkMascB female to male ratios relative to male copy numbers corresponding to the autosomal hypothesis (both EkMasc and EkMascB located on autosomes; F:M ratio = 1.00), and the Z chromosomal hypothesis (F:M ratio = 0.50). Error bars indicate standard deviation. (TIF) [file pgen.1009420.s004.tif]

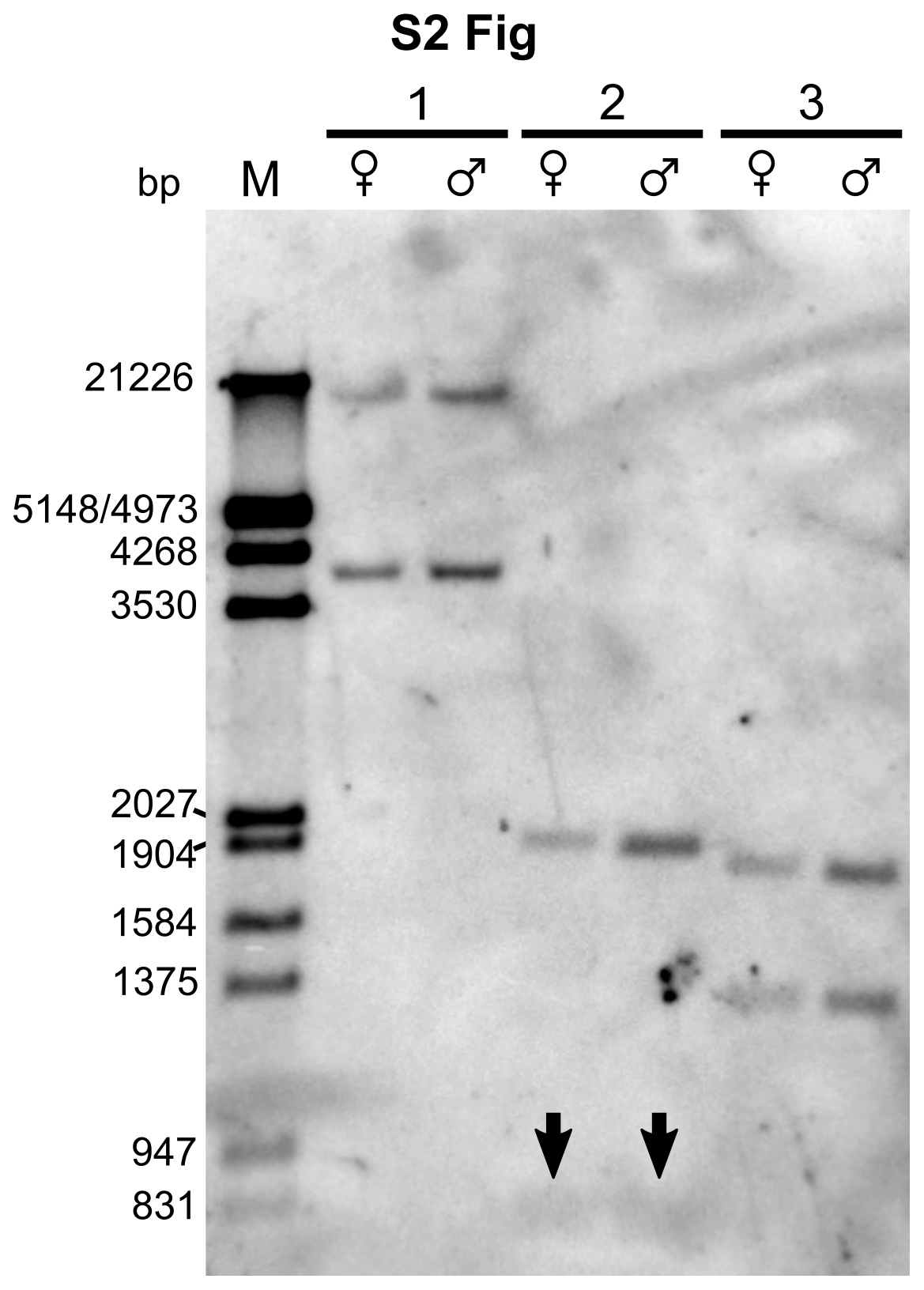

Supplement: S2 Fig — Two signals can be identified in the genomic DNA of both female and male samples double digested with (1) NdeI x NotI, (2) DraI x NheI, and (3) AgeI x BspHI. Arrows indicate highly diffused bands. Note that female signals are weaker than male signals. Indicated are the marker (M) in bp, female (♀) and male (♂) samples. (TIF) [file pgen.1009420.s005.tif]

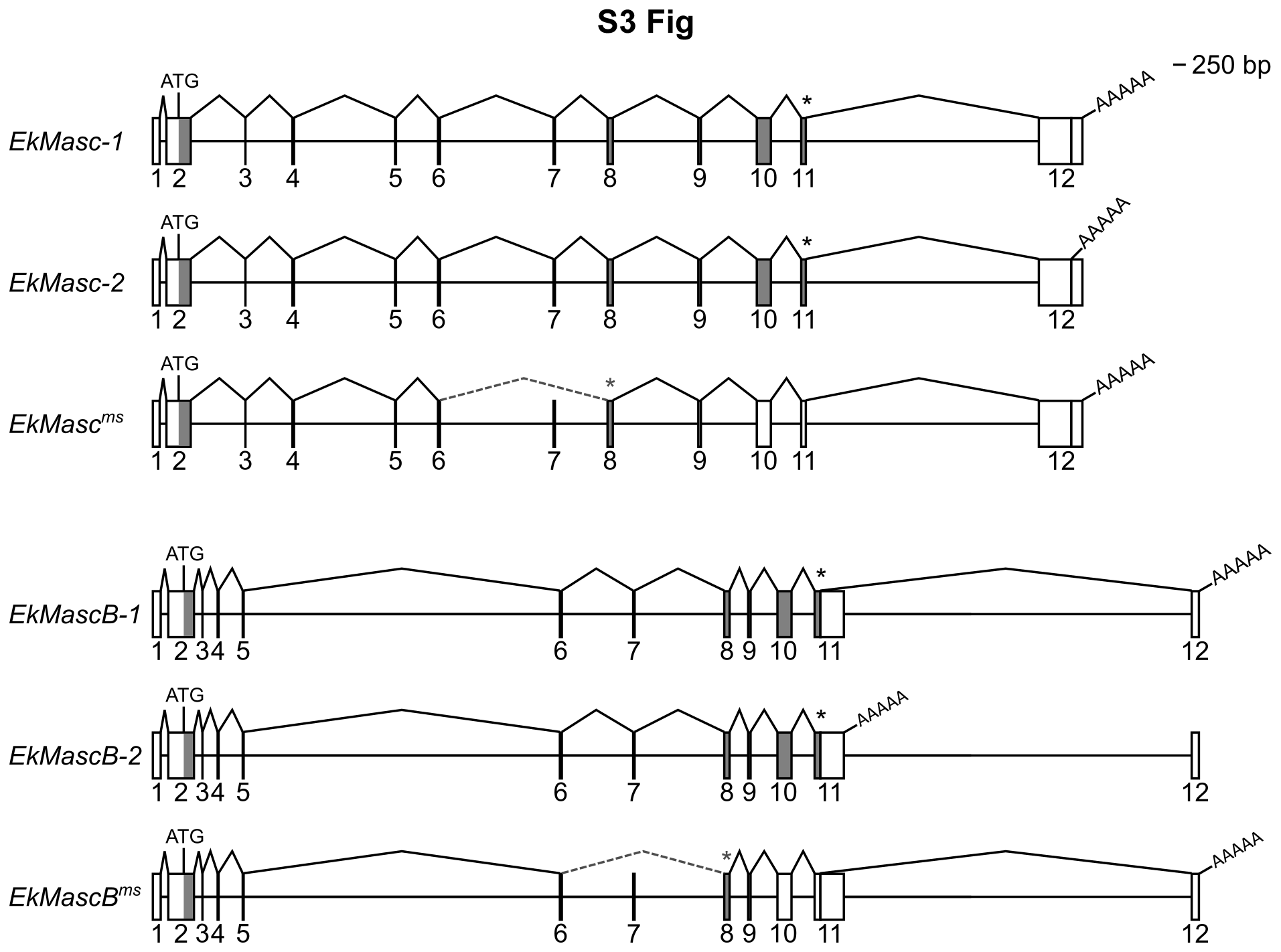

Supplement: S3 Fig — Two poly-adenylation sites are indicated for EkMasc, i.e. EkMasc-1 and EkMasc-2, as well as the open reading frame (grey) including the start (ATG) and stop codon (*). Additionally shown is the splice variant EkMascms skipping exon VII (dashed grey line) and its corresponding premature stop codon (grey *). Similar splice patterns were observed for the EkMascB gene. (TIF) [file pgen.1009420.s006.tif]

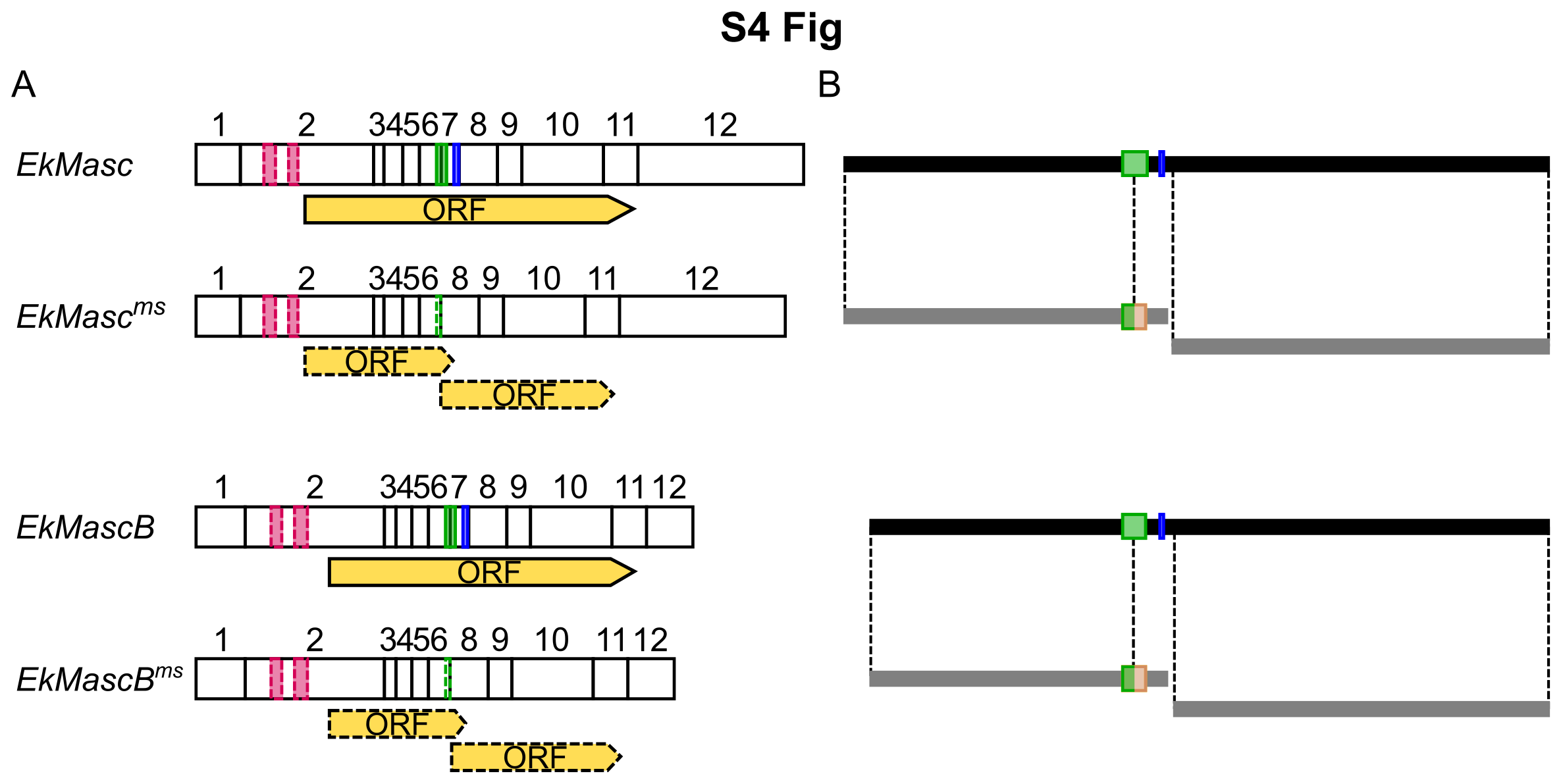

Supplement: S4 Fig — (A) Schematic drawings of the main alternative splice forms of EkMasc and EkMascB. Indicated are the exons (white boxes), the degenerated zinc finger motifs (dashed pink boxes), the bipartite nuclear localization signal (green box), the masculinizing domain (blue box), and the open reading frame (ORF). Note that the masculinizing domain is absent in EkMascms and EkMascBms splice variants and that there are two potential ORFs for these splice forms. (B) Schematic representation of the proteins translated from each of the transcripts (in black or grey) with the same domains indicated as in A. The first of the two potential EkMascms proteins is identical to the main EkMasc protein for the region indicated by dashed lines. This potential protein also contains a complete bipartite nuclear localization signal (green/brown box), the first half of which is identical to the EkMasc protein. The second potential EkMascms protein is a short version of the EkMasc protein sharing complete amino acid identity with the C-terminus of the EkMasc protein, except for the first residue. The same pattern is observed for EkMascB and potential EkMascBms proteins. (TIF) [file pgen.1009420.s007.tif]

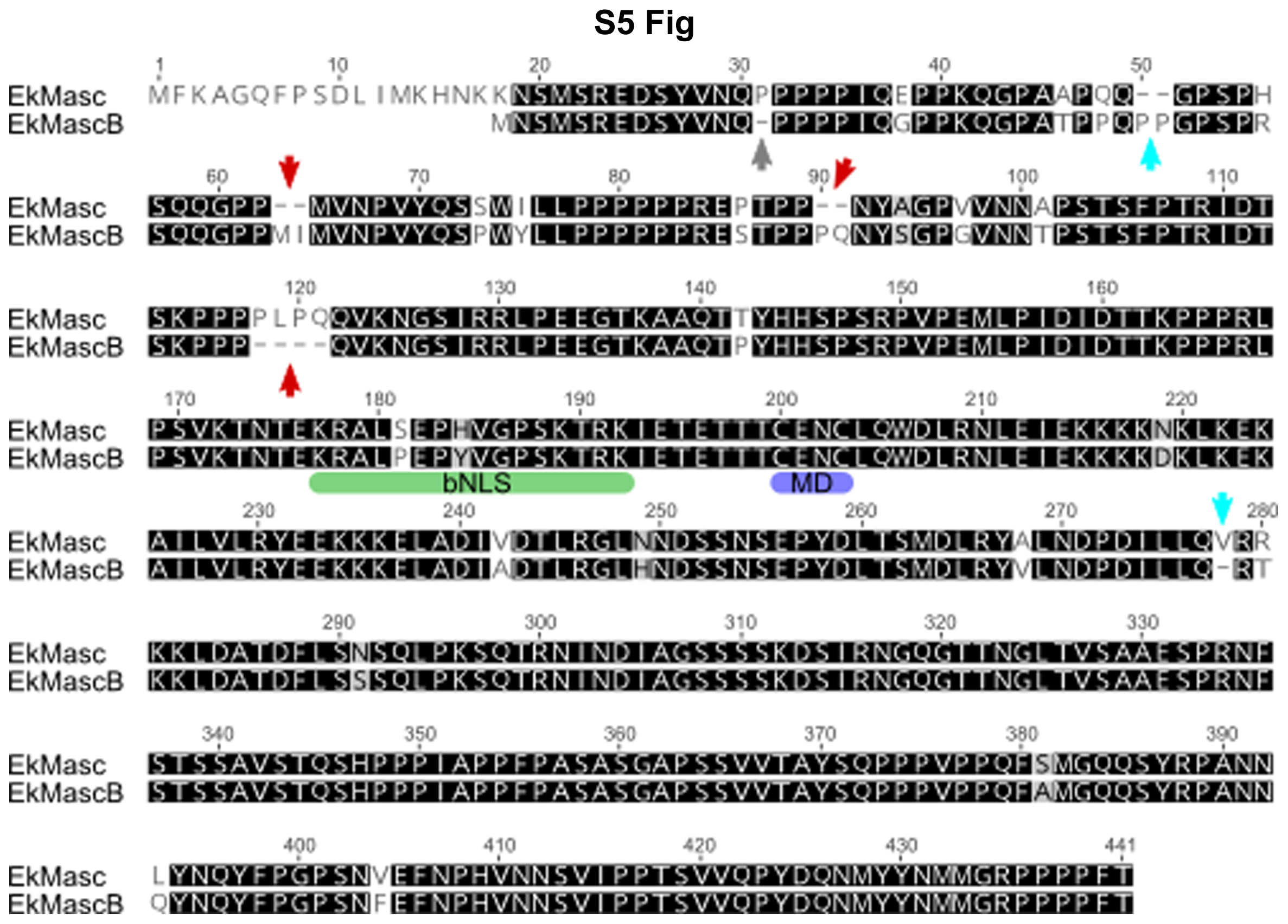

Supplement: S5 Fig — Indicated in the alignment are the conserved bipartite nuclear localization signal (bNLS; green) and the masculinizing domain (MD; blue). Also indicated are deletions (red arrows) and insertions (cyan arrows) in either EkMasc or EkMascB based on comparison to the Masc protein sequence of the closely related Plodia interpunctella. A single grey arrow (at amino acid 31) indicates an indel between EkMasc and EkMascB that cannot be categorized as a deletion or an insertion in either protein sequence based on the Masc protein sequence of P. interpunctella. (TIF) [file pgen.1009420.s008.tif]

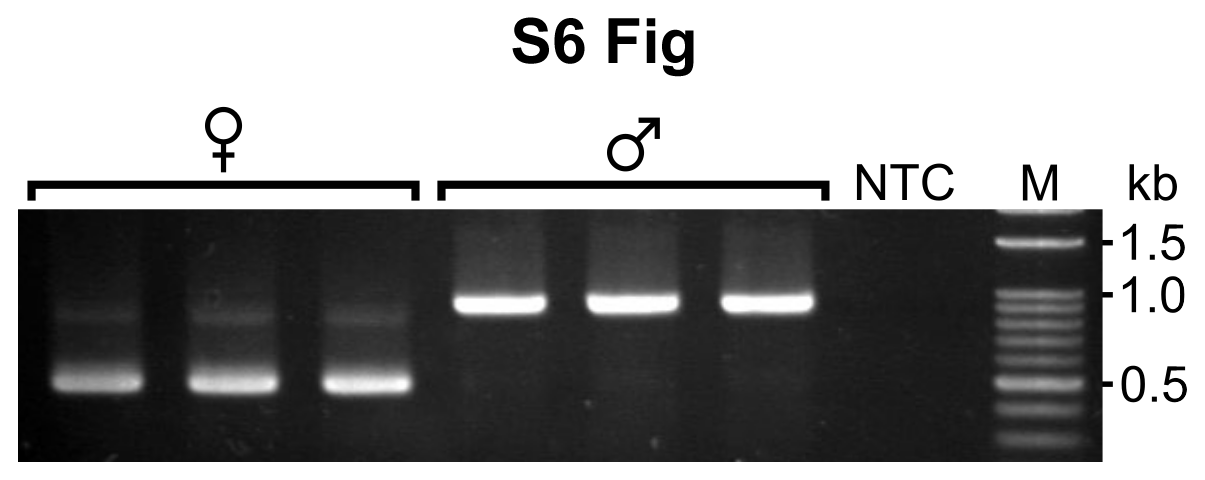

Supplement: S6 Fig — Note the two bands in female samples, the lower band at approximately 500 bp corresponding to the degenerated EkMasc copies identified on the W chromosome. Indicated are marker (M) in kb, no template control (NTC), female (♀) and male (♂) samples. (TIF) [file pgen.1009420.s009.tif]

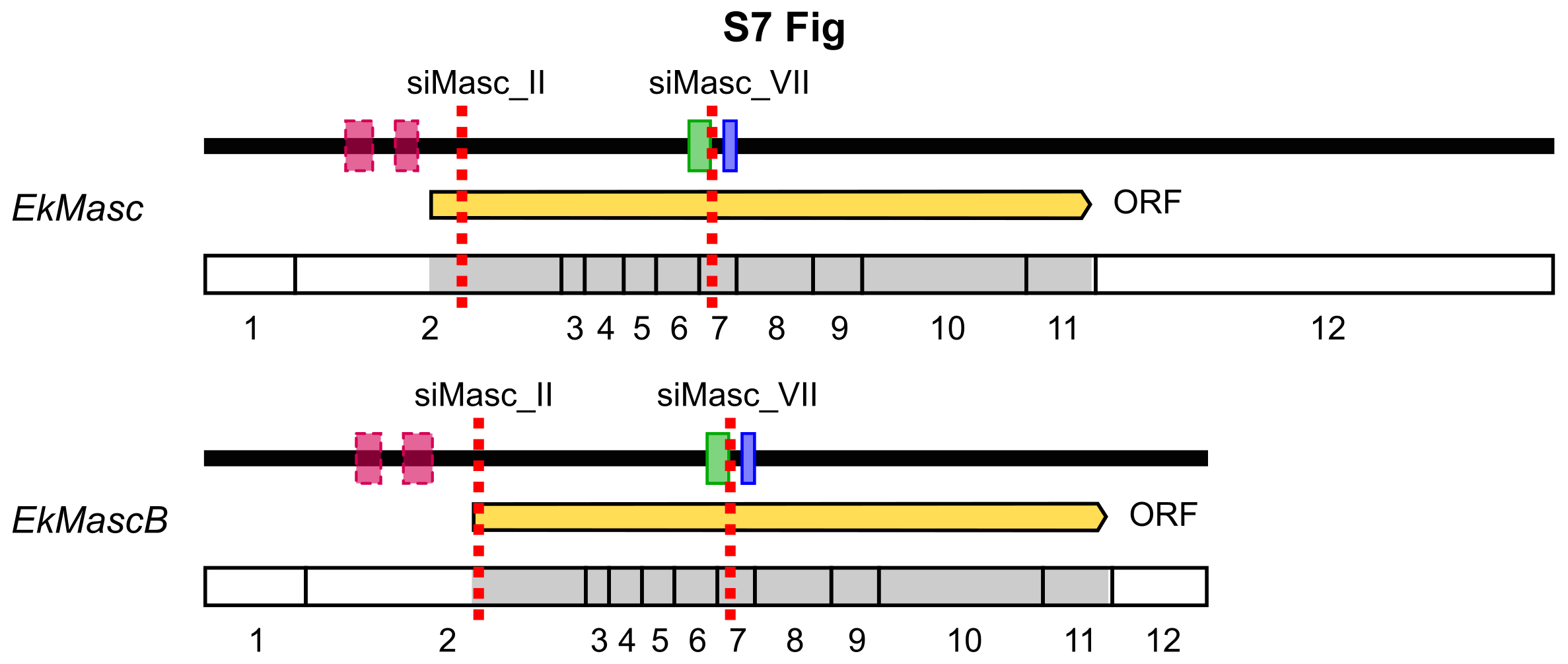

Supplement: S7 Fig — Indicated in the figure are the degenerated zinc finger motifs (pink boxes) upstream of the open reading frame, the bipartite nuclear localization signal (green box), the male determining region (blue box), the open reading frame (yellow) and the two siRNAs targeting EkMasc and EkMascB (red dashed lines). Also shown is an exon representation of the genes. (TIF) [file pgen.1009420.s010.tif]

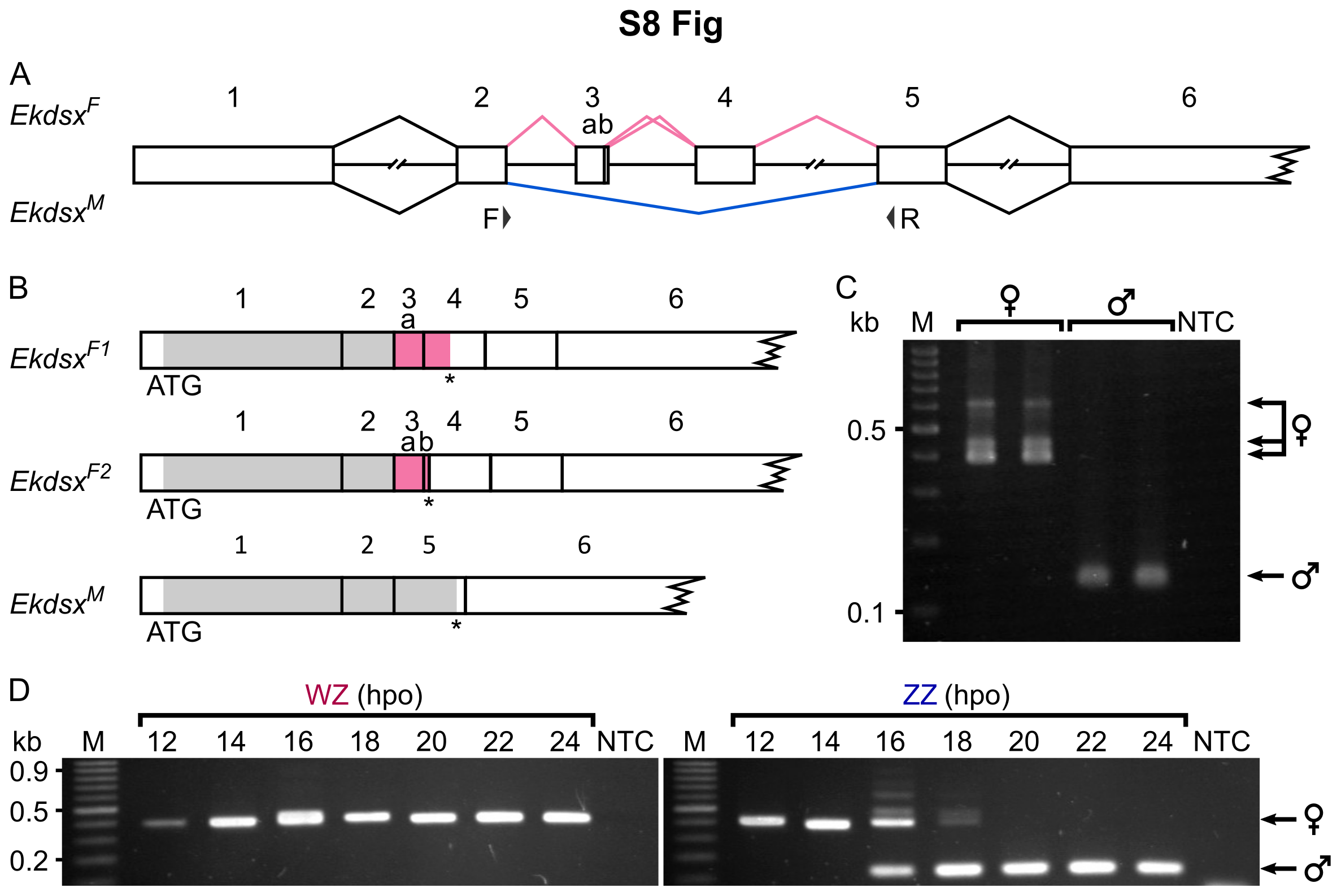

Supplement: S8 Fig — (A) Schematic representation of female- (pink line) and male-specific (blue line) splicing patterns of Ekdsx. Indicated below the figure are the targets of the primers used throughout the article (F and R). (B) The two dominant female- and the dominant male-specific transcripts and their predicted respective open reading frames (in grey/pink). (C) Sex-specific splicing of Ekdsx as detected by the primers indicated in A. A currently uncharacterized third female-specific splice variant is also visible (the top arrow). (D) Sex-specific splicing pattern of Ekdsx during early development in WZ (left) and ZZ (right) individuals. Note transitions of Ekdsx splicing from female-specific to male-specific in ZZ individuals only, 16–18 hours post oviposition (hpo). Indicated are marker (M) in kb, and no template control (NTC). (TIF) [file pgen.1009420.s011.tif]

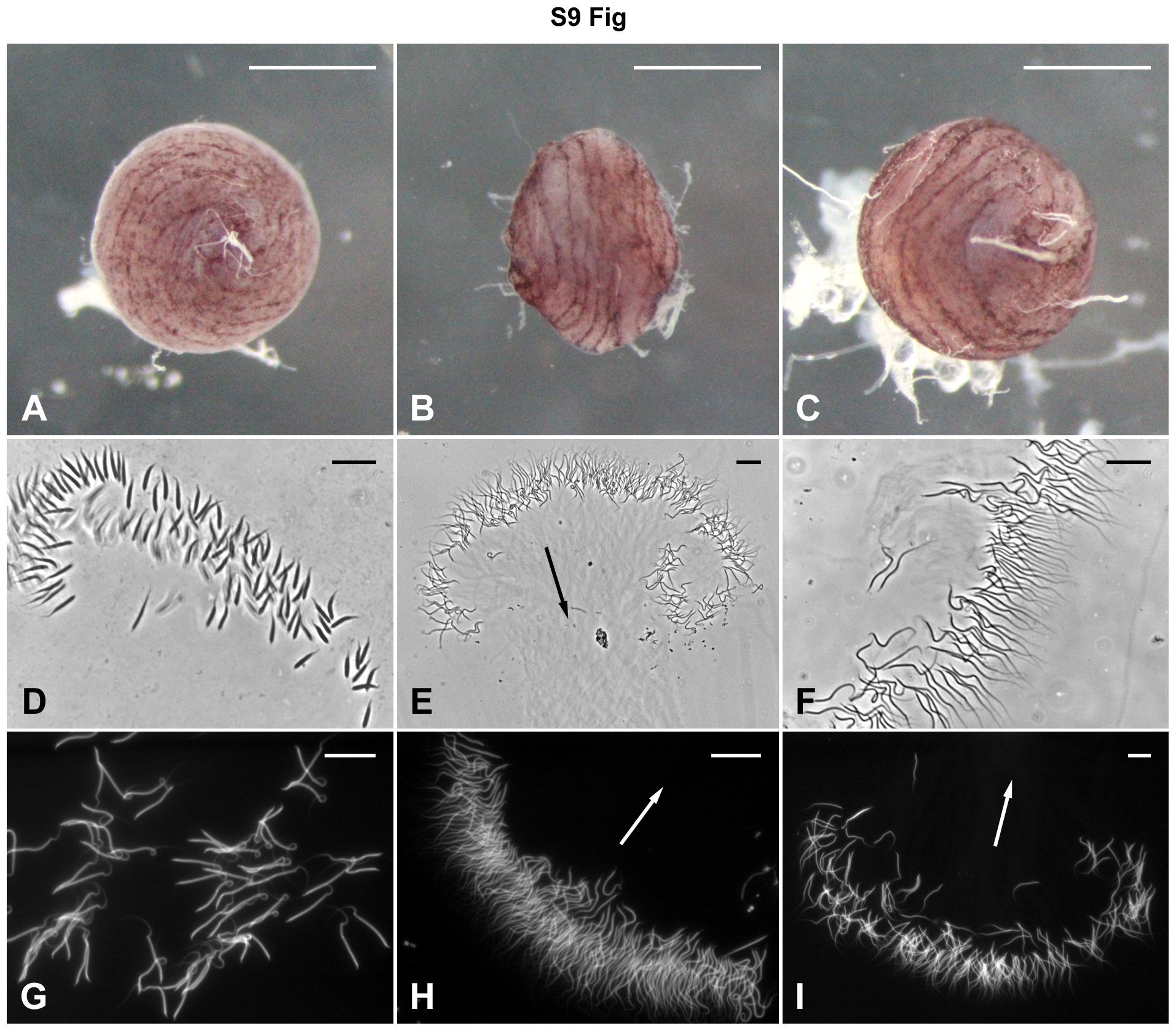

Supplement: S9 Fig — (A, D, G) Control: 4 males of the WT-C02 strain examined. (B, E, H) Injection with siMasc_VII: 5 males stored in the freezer examined. (C, F, I) Injection with siMasc_II: 3 males stored in the freezer examined. (A–C) Images of dissected testes in physiological solution. Bar = 0.5 mm. (D–F) Nuclei of eupyrene sperm stained with lactic acetic orcein observed using a phase contrast microscope. Bar = 10 μm. (G–I) Nuclei of eupyrene sperm stained with DAPI (4’,6-diamidino-2-phenylindole) observed using a fluorescence microscope. Bar = 10 μm. D and F show part of the sperm bundle head; E, H, and I, show the complete head of the sperm bundle (arrows indicate directions of sperm tails); G shows spread nuclei of eupyrene sperm. (TIF) [file pgen.1009420.s012.tif]

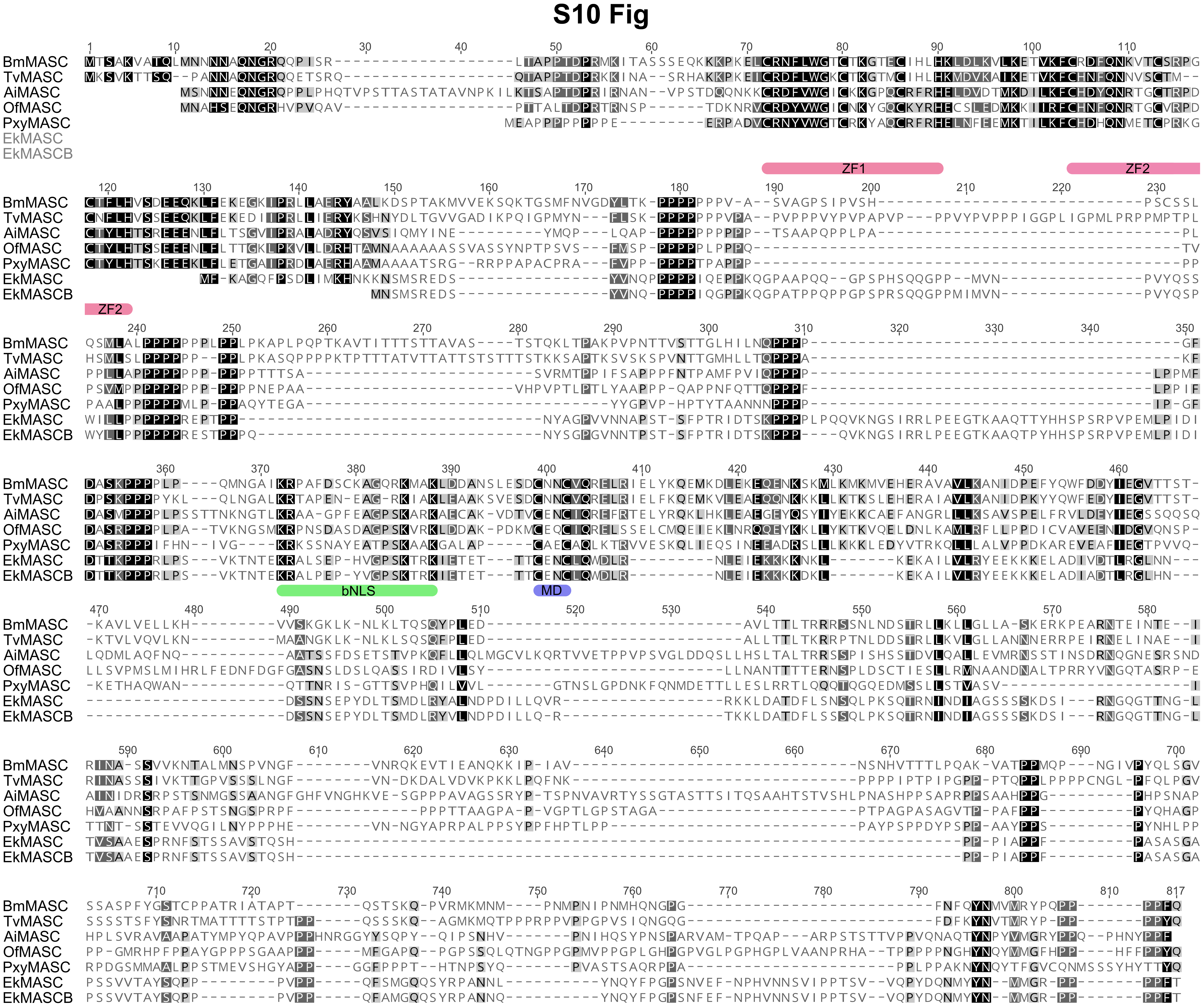

Supplement: S10 Fig — The alignment shows low levels of amino acid identity even within the functional domains. Indicated below the alignment are the conserved zinc finger domains (ZF1 & ZF2; pink), the bipartite nuclear localization signal (bNLS; green) and the masculinizing domain (MD; blue). Aligned are the Masc protein sequences of Bombyx mori (Bm), Trilocha varians (Tv), Agrotis ipsilon (Ai), Ostrinia furnacalis (Of), Plutella xylostella (Pxy), and Ephestia kuehniella (Ek). (TIF) [file pgen.1009420.s013.tif]

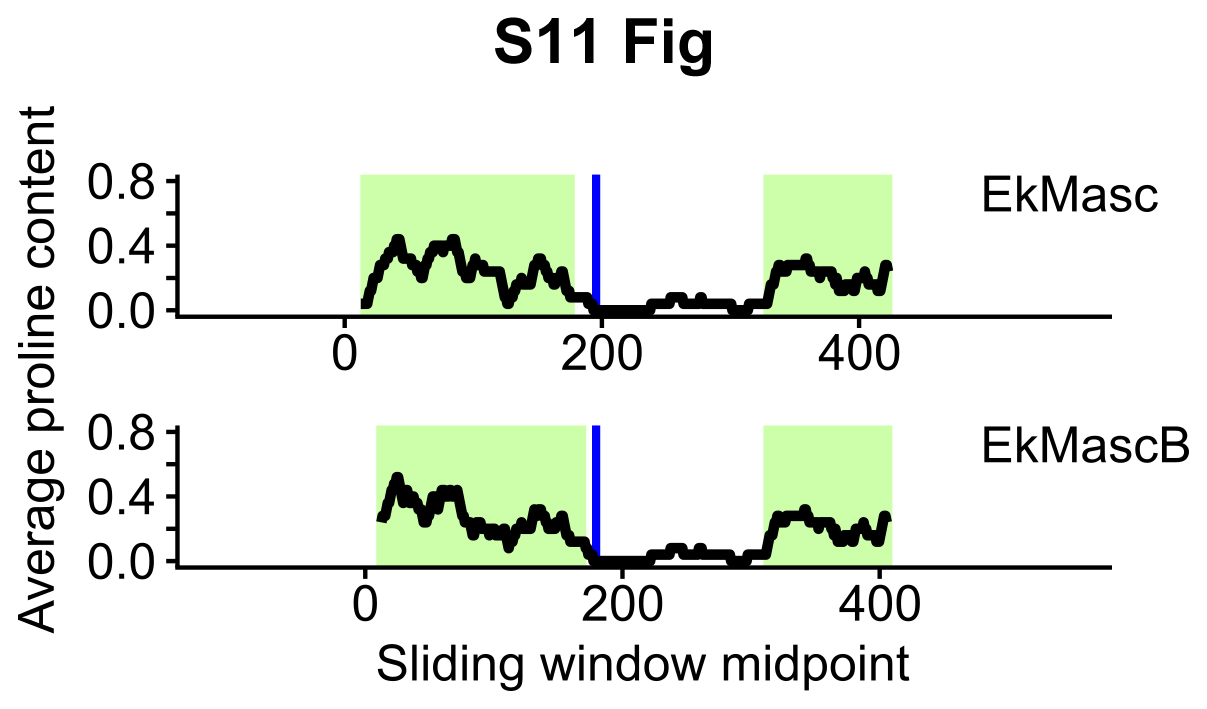

Supplement: S11 Fig — Note that the proline distribution in both proteins is very similar. (TIF) [file pgen.1009420.s014.tif]

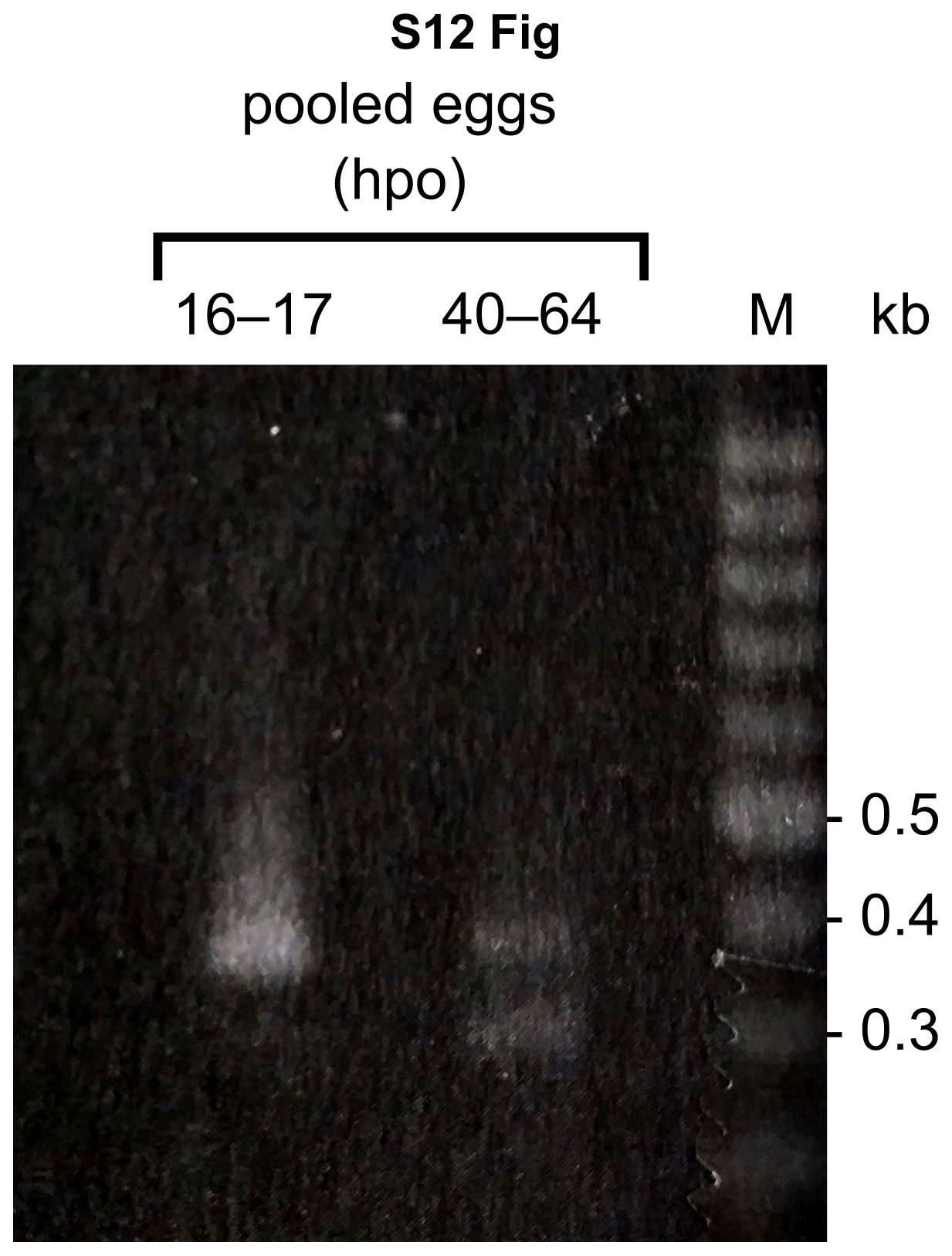

Supplement: S12 Fig — RT-PCR using cDNA obtained from pooled unsexed eggs samples of 16–17 hours post oviposition (hpo) and 40–64 hpo. At 16–17 hpo, only the splice variant containing exon 7 is visible, whereas at 40–64 hpo an additional band corresponding to the EkMascms and EkMascBms splice variants is visible. Indicated is marker (M) in base pairs (bp). (TIF) [file pgen.1009420.s015.tif]

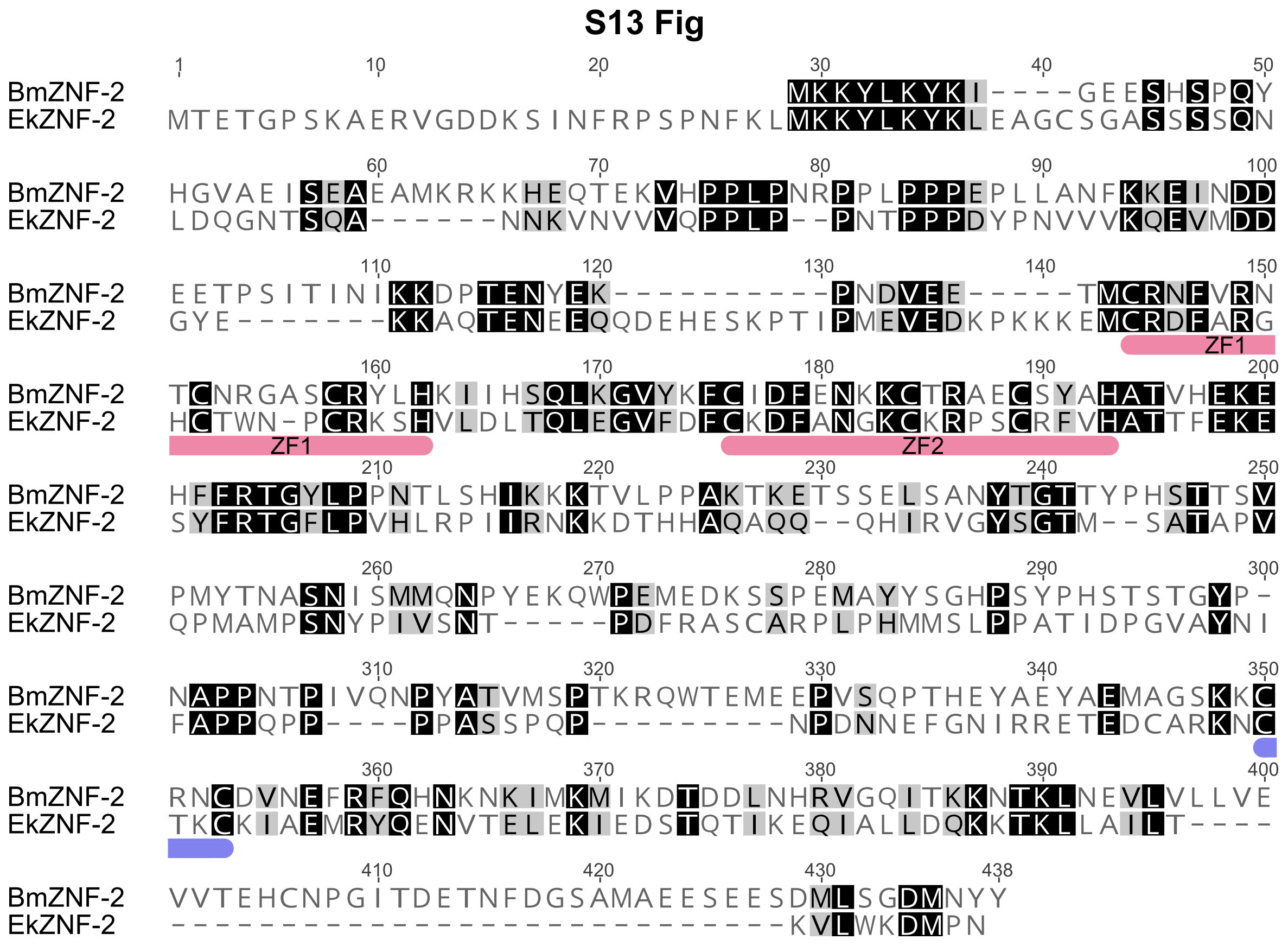

Supplement: S13 Fig — Indicated in pink are the two zinc finger domains (ZF1 and ZF2), and in blue two cysteine amino acids separated by two amino acids similar to the masculinizing domain in lepidopteran Masc proteins. (TIF) [file pgen.1009420.s016.tif]
