# Supplementary material for: A conserved role of the duplicated Masculinizer gene in sex determination of the Mediterranean flour moth, Ephestia kuehniella
Source: PLoS Genet. 2021 Aug 2;17(8):e1009420. doi: 10.1371/journal.pgen.1009420 (PMC8360546; doi:10.1371/journal.pgen.1009420)
Supplement: S1 Methods — Identification and isolation of EkMasc and EkMascB sequences. Assessment of copy number and localization of Masc using Southern hybridization. Z‐linkage of EkMasc and EkMascB by quantitative real‐time PCR (qPCR). Tissue‐specific splicing of Masc. Expression analysis of EkMasc and EkMascB during embryogenesis. Back extraction DNA isolation protocol. Functional analysis of EkMasc and EkMascB. (PDF) [file pgen.1009420.s017.pdf]

S1 Methods

Identification and isolation of *EkMasc* and *EkMascB* sequences

Cloning, plasmid isolation and sequencing. The PCR products were pooled (eggs and pupa samples separate) and purified using the Wizard SV Gel and PCR Clean-Up System (Promega, Madison, WI, USA) and subsequently cloned by ligating overnight into the pGEM-T Easy vector (Promega) and transforming into *Escherichia coli* strain DH5α according to the manufacturer's protocol with minor adjustments. Cells were heat-shocked for 90 s at 42°C, and only 800 µL of LB medium was added during the recovery phase. Positive colonies were screened by PCR using universal M13 primers, and amplicons were checked on a 1% agarose gel. Colonies with inserts of the predicted size were inoculated into 3 mL of LB medium containing 50 µL/mL ampicillin and incubated overnight at 37°C, 200 rpm. Plasmids were subsequently isolated using the NucleoSpin Plasmid kit (Macherey-Nagel, Düren, Germany) according to the manufacturer's protocol and inserts were sequenced (SEQme, Dobříš, Czech Republic).

RACE-PCR. The first round of 3'-RACE-PCR was performed with a gene-specific forward primer (Masc_F1) and the abridged universal amplification primer (AUAP). This 10 µL final PCR reaction mixture was composed of 2 µM gene-specific forward primer, 0.2 µM AUAP, 0.8 mM dNTPs, 1× Ex *Taq* DNA polymerase buffer, 0.25 units of Ex *Taq* DNA polymerase and 1 µL of template cDNA. The primer concentration of the forward primer was increased with respect to the reverse primer to avoid off-target amplification of only the reverse primer. An additional round of semi-nested PCR was performed to increase gene-specific amplification. The PCR reaction volume was increased to 20 µL and the forward primer was substituted with a nested gene-specific primer (Masc_VII_F1) at a final concentration of 0.2 µM (equal to the reverse primer). In addition, the amount of Ex *Taq* DNA polymerase was increased to 0.5 units per reaction. Template for the semi-nested PCR was 100× diluted non-purified PCR product of the preceding 3' RACE-PCR. Both PCR reactions were performed at 94°C for 3 min; 35 cycles of 94°C for 40 s, 55°C for 40 s and 72°C for 2 min 30 s; and a final extension at 72°C for 3 min. Final PCR products were visualized on a 1% agarose TAE gel using ethidium bromide (EtBr) as a stain. PCR products were purified directly or strong bands were cut from the gel and purified using Wizard SV Gel and PCR Clean-Up System (Promega) as described by the manufacturer, cloned and sequenced as described above.

For 5'-RACE-PCR, we followed the procedure described in Frohman et al. (1988). The synthesized cDNA was purified using an Illustra Sephadex G-50 column (GE Healthcare Life Sciences, Buckinghamshire, UK) and then poly-A tail was added using Terminal Transferase (New England Biolabs, Ipswich, MA, USA) according to the manufacturer's protocol with 200 µM dATPs and an incubation time of 45 min. Amplification of gene specific 5' RACE transcripts was done in two steps. The first step was performed in a 10 µL PCR volume and was composed of 0.2 µM adapter primer (AP), 0.2 µM gene-specific primer Masc_IV_R1, 0.8 mM dNTPs, 1× Ex *Taq* DNA polymerase buffer, 0.25 units of Ex *Taq* DNA polymerase and approximately 10-30 ng of poly-A-tailed cDNA. For the second step, PCR volumes were increased to 20 µL, primers were substituted with AUAP and the nested gene-specific primer Masc_R1, and 1 µL of the 10× diluted PCR-product from the previous step was used as template DNA. Both thermocycling reactions were performed as described above for 3' RACE PCR. Final PCR products were processed as described for 3' RACE-PCR.

Frohman MA, Dush MK, Martin GR. Rapid production of full-length cDNAs from rare transcripts: amplification using a single gene-specific oligonucleotide primer. Proc Natl Acad Sci U S A. 1988; 85(23):8998–9002. doi: 10.1073/pnas.85.23.8998

Assessment of copy number and localization of *Masc* using Southern hybridization

DNA digestion for Southern hybridization. DNA was double digested using *Nde*I × *Not*I, *Dra*I × *Nhe*I (all Fermentas, Vilnius, Lithuania), or *Age*I × *Bsp*HI (New England Biolabs) following the manufacturer's instructions, using Orange buffer, Tango buffer, or CutSmart buffer, respectively. Digestions were incubated at 37°C overnight and an additional volume of these enzymes was added the next morning, after which the samples were incubated for another 1 h to ensure complete digestion of the DNA. Digestion reactions were stopped by adding Gel Loading Dye Purple (New England Biolabs) to a final 1× concentration. Then the digested DNA was separated on a 1% TBE agarose gel, run at 70 V per centimeter of gel distance for 4 h.

Southern hybridization probe. Primers for the Southern hybridization probe were designed in the region of high sequence identity between *EkMasc* and *EkMascB* targeting exon XI using Geneious 9.1.6. Initial PCR was performed using Masc_Sb_F and Masc_Sb_R primers and male genomic DNA as a template using the standard PCR mix described above (see "Identification and isolation of *EkMasc* and *EkMascB* sequences"). The obtained PCR products were checked on a 1% agarose gel for successful amplification, purified, cloned, and sequenced as described above. Plasmid DNA was isolated from a clone containing the expected *EkMasc* sequence and this plasmid DNA was used in PCR to generate a template for PCR-labeling. This 50 µL PCR reaction consisted of 0.2 µM of both Masc_Sb_F and Masc_Sb_R, 0.2 mM dNTPs, 1× Ex *Taq* Buffer, 0.25 units of Ex *Taq* DNA polymerase, and 5 ng of plasmid DNA. The reaction was performed using the thermocycling program described for the initial isolation of *EkMasc* and *EkMascB*. Products were purified using the Wizard SV Gel and PCR Clean-Up System according the manufacturer's instructions and used as a template in the labeling reaction. The PCR-labeling reaction consisted of 400 nM each of Masc_Sb_F and Masc_Sb_R, 40 µM each of dATP, dCTP and dGTP, 14.4 µM dTTP, 25.6 µM of digoxigenin-11-dUTPs (Roche Diagnostics, Mannheim, Germany), 1× Ex *Taq* Buffer, 0.625 units of Ex *Taq* polymerase, and approximately 5 ng of template in a total reaction volume of 25 µL. Amplification of the probe was done according to the following profile: denaturation at 94°C for 90 s (1 cycle), denaturation at 94°C for 30 s, annealing at 55°C for 30 s, elongation at 72°C for 60 s (35 cycles), and final elongation at 72°C for 60 s (1 cycle). The probes were then purified using an Illustra Sephadex G-50 column, and their concentrations were measured on a Qubit 3.0 Fluorometer using the dsDNA BR Assay Kit (Invitrogen, Carlsbad, CA).

Z-linkage of *EkMasc* and *EkMascB* by quantitative real-time PCR (qPCR)

qPCR. Concentrations of DNA extracted from *Ephestia kuehniella* larvae were measured on an Invitrogen Qubit 3.0 Fluorometer using the dsDNA BR Assay Kit. Primers were designed to target both *EkMasc* and *EkMascB* simultaneously in a conserved region of the genes using Geneious 9.1.6. The protein sequence of *Bombyx mori* acetylcholinesterase type 2 (accession number ABY50089.1) was used in a tBLASTn search against the *E. kuehniella* genome to identify a partial *EkAce-2* sequence (accession number MW505944), which was then used to design primers using Geneious 9.1.6. qPCR was carried out on a C1000 Thermal cycler CFX96 Real-Time System (Bio-Rad, Hercules, CA) with a cycle program of 95°C for 3 min initial denaturation followed by 45 cycles of 94°C for 30 s denaturation, 60°C for 20 s combined annealing and extension, 95°C for 15 s final denaturation, and 65°C to 95°C by 0.5°C steps of 5 s to analyze the melting curve. Experiments were run in FrameStar 96 well plates sealed with qPCR adhesive foil (both Institute of Applied Biotechnologies, Prague, Czech Republic). Primer efficiencies were determined by dilution series analysis and calculated using the Bio-Rad CFX Manager 3.0 software (Bio-Rad Laboratories, Hercules, CA).

Tissue-specific splicing of *Masc*

Primer design. Primers were designed using Geneious 9.1.6 with "Product Size" setting to 450–500 for *Cydia pomonella* and 1000–1200 for *Plodia interpunctella*. To ensure the design of primers that would amplify both *Masc* and *Masc*^{ms} splice variants, the "Target Region" setting was set to include the exon containing the masculinizing region in both species. To this end, the *CpMasc* sequence was manually assembled from the non-assembled transcriptome reads, as the tBLASTn search against the assembled transcriptome did not yield any significant hits. An initial fragment of *CpMasc* was obtained by a tBLASTn search against the non-assembled *C. pomonella* 1-day-old eggs transcriptome reads (accession number SRX5284305), after which the initial sequence was extended using multiple rounds of BLASTn searches until an open reading frame was detected (accession number MW505945).

Dissection. We dissected out the testes of the pre-final instar larvae (prior to fusion of the testes) of *E. kuehniella*, *P. interpunctella*, and *C. pomonella* in physiological solution (Glaser 1917; cited in Lockwood, 1961). For each individual, we immediately isolated RNA from the testes and separately from the remaining body tissue using TRI Reagent as described in Materials and methods (see main text). For testis samples, 20 µg of RNA grade glycogen (Thermo Fisher Scientific, Waltham, MA) was added before precipitation to reduce loss of RNA. In *E. kuehniella*, the same procedure was repeated for pupa samples.

Lockwood AP. 'Ringer" solutions and some notes on the physiological basis of their ionic composition. Comp Biochem Physiol. 1961; 2:241–289. doi: 10.1016/0010-406x(61)90113-x

Expression analysis of *EkMasc* and *EkMascB* during embryogenesis

Sample treatment. For each time point, sixteen embryos were crushed individually in TRI Reagent and stored at –80°C until RNA was isolated to ensure a minimum of three samples for each sex would be available to measure expression levels. Prior to precipitation, 20 µg glycogen RNA grade (Thermo Fisher Scientific) was added as co-precipitate to reduce RNA loss. RNA pellets were stored at –80°C in ethanol until further use.

Back extraction DNA isolation protocol.
https://www.thermofisher.com/cz/en/home/references/protocols/nucleic-acid-purification-and-analysis/dna-extraction-protocols/tri-reagent-dna-protein-isolation-protocol.html
In short, an equal volume of back extraction buffer (4 M guanidine thiocyanate, 50 mM sodium citrate, 1 M Tris base) was added to the organic phase, samples were mixed for 15 s, centrifuged, and the aqueous phase was transferred to a new tube. 20 µg of glycogen was added to the samples and DNA was precipitated by adding 2/3 volume of isopropanol and incubating for 5 min at room temperature. After centrifugation, the pellets were washed twice with 70% ethanol and dissolved in 20 µL nuclease-free water.

Identification of *Ekrp49* and primer design. A set of degenerated primers (rp49_deg_F1 and rp49_deg_R1) was designed for *rp49* in a conserved region of the gene in Lepidoptera based on sequences from *B. mori* (NM_001098282.1), *Glyphodes pyloalis* (MH715949.1), *Helicoverpa armigera* (JQ744274.1), and *Heliconius melpomene* (EF207973.1). The PCR mixture and cycling program were the same as described for the initial isolation of *EkMasc* and *EkMascB*, but with an annealing temperature of 52°C and scaled up to a final volume of 40 µL. The products obtained were purified, cloned and sequenced as described above. Primers for *Ekrp49* (qrp49_F2 and qrp49_R2) were

designed on the *E. kuehniella* sequence obtained (accession number MW505943) using Geneious 9.1.6, while primers for *EkMasc* (qMasc_F2 and qMasc_R2) and *EkMascB* (qMascB_F1 and qMascB_R1) were designed manually in highly diverged regions of the genes. Manually designed primers were subsequently checked for primer-dimers, hairpins, and off-target amplification using Geneious 9.1.6. All primers were tested with single embryo samples and pooled embryo samples for potential off-target amplification and primer-dimers prior to the experiment. To detect off-target amplification and primer-dimers, melting curves were analyzed for secondary peaks and products were run on a 1.5% agarose gel. In addition, amplification efficiencies of each primer pair were determined by a three-fold dilution series of the pooled embryos cDNA sample using the Bio-Rad CFX Manager 3.0 software (Bio-Rad Laboratories).

Functional analysis of *EkMasc* and *EkMascB*

siRNA design. *EkMasc* and *EkMascB* coding sequences were aligned and screened for two consecutive adenine nucleotides, conserved in both sequences, in exons II and VII. For both exons, additional consecutive 19 nucleotides of perfect homology between the two sequences were selected as potential siRNA target sequences and were compared against the *E. kuehniella* genome using BLASTn to identify potential off-target binding sites. For both siRNA duplexes, the lowest homology to any other sequence in the genome (<15 nt perfect homology), GC-content, and sequence asymmetry in the siRNA duplexes were assessed.

Egg collection and microinjection. Freshly emerged adult females and males were collected and left to mate overnight. Mating couples were isolated the next morning. Because *E. kuehniella* females lay eggs at dusk, the females were transferred to a Petri dish approximately 5 min before the lights off and eggs were collected by 45 min later. Glass microscope slides were prepared by placing a wet piece of filter paper near each end of the slide, and eggs were aligned against the paper using a slightly wetted paint brush. Injections were done using a FemtoJet Microinjector (Eppendorf, Hamburg, Germany). Needles were prepared from 1 mm outer diameter, 0.58 mm inner diameter borosilicate glass capillaries with filament (Sutter Instrument, Novato, CA, USA) using a Magnetic Glass Microelectrode Horizontal Needle Puller PN-31 (Narishige, Tokyo, Japan), as described in Kotwica-Rolinska et al. (2019). After injection, filter papers were removed, and the glass slides were transferred to a Petri dish containing wetted tissue paper to keep high humidity levels. Petri dishes were incubated at 21–22°C.

Kotwica-Rolinska J, Chodakova L, Chvalova D, Kristofova L, Fenclova I, Provaznik J, et al. CRISPR/Cas9 genome editing Introduction and optimization in the non-model insect *Pyrrhocoris apterus*. Front Physiol. 2019; 10:891. doi: 10.3389/fphys.2019.00891

Identification of *Ekdsx*. To identify a *dsx* ortholog in *E. kuehniella*, we used the male DSX protein from *B. mori* (BmDSXM; accession number AHF81625.1) to perform a tBLASTn search against the *E. kuehniella* genome. However, the male-specific protein segment did not provide any significant hits. Therefore, we used BmDSXM to perform a BLASTp search against all lepidopteran species and obtained the predicted DSXM protein of the closely related *Amyelois transitella* (accession number XP_013184257.1). This AtDSXM sequence was subsequently used to identify the male-specific segment of *Ekdsx*.
